# Advances in the study of death receptor 5

**DOI:** 10.3389/fphar.2025.1549808

**Published:** 2025-03-12

**Authors:** Xuan Qiao, Shuang Guo, Zhiyun Meng, Hui Gan, Zhuona Wu, Yunbo Sun, Shuchen Liu, Guifang Dou, Ruolan Gu

**Affiliations:** ^1^ Graduate Collaborative Training Base of Academy of Military Medical Sciences, Hengyang Medical School, University of South China, Hengyang, China; ^2^ Beijing Institute of Radiation Medicine, Beijing, China

**Keywords:** death receptor 5, TRAIL-DR5 signaling pathway, DR5 agonist, DR5 antagonist, tumors, cardiovascular disease, autoimmune diseases, radiation damage protection

## Abstract

DR5, a receptor with the highest affinity for TRAIL under physiological conditions, selectively induces apoptosis in specific target cells such as tumor and aberrant immune cells, while minimally affecting normal cells. The TRAIL-DR5 signaling pathway is a crucial regulatory mechanism when the body responds to various exogenous interference factors, including viruses, chemicals, and radiation. This pathway plays a vital role in maintaining physiological homeostasis and in the pathological development of various diseases. Different modulations of DR5, such as upregulation, activation, and antagonism, hold significant potential for therapeutic applications in tumors, cardiovascular diseases, autoimmune diseases, viral infections, and radiation injuries. This article provides an overview of the current research progress on DR5, including the status and prospects of its clinical applications.

## 1 Introduction

Tumor necrosis factor-related apoptosis-inducing ligand (TRAIL), a member of the TNF superfamily, is expressed in most human cells and primarily induces apoptosis in various cancer cell lines without harming normal cells. Its receptors include death receptor (DR) 4, DR5, death decoy receptor (DcR) I, DcR2, and the soluble receptor osteoprotegerin. DR5, under physiological conditions, demonstrates the strongest affinity for TRAIL. The TRAIL-DR5 signaling pathway is a major regulatory pathway when the body responds to diverse exogenous stimuli, playing an essential role in both physiological homeostasis and disease development. Studies have shown that DR5 protein expression is significantly upregulated in various disease-target organs. Through the modulation of DR5 expression and the intervention with DR5 activators or antagonists, significant therapeutic potential has been demonstrated for treating tumors, cardiovascular diseases, autoimmune diseases, severe viral infections, and radiation injuries. DR5 has emerged as a focal point for clinical disease treatment research. This paper reviews the basic features, main physiological functions, and significant current research progress on DR5, including the status and prospects of its clinical application research.

## 2 Overview of DR5

### 2.1 Structure and properties of DR5

Known by multiple names including TRAIL-R2, TNFRSF10B, CD262, Apo2, Killer/Ly98, TRICK2A, and TRICKB, DR5 is a type I transmembrane protein. It consists of a signal peptide, an extracellular domain, a transmembrane domain, and an intracellular domain. The full-length DR5 cDNA is 1,146 bp, encoding 381 amino acids (Mert and Sanlioglu, 2017; Min et al., 2019). Gene transcription of DR5 occurs at 8q21.3, with a total DNA sequence length of 49,055 base pairs and a DR5 transcript length of 4,154 nucleotides (Mert and Sanlioglu, 2017). The DR5 mRNA 3′-UTR region encompasses 2,538 nucleotides, constituting more than half of the entire transcript.

Although DR5 and DR4, another major activating TRAIL apoptosis receptor, have relatively high homology (Chaudhary et al., 1997; Wiley et al., 1995) in the cysteine-rich domain and the death domain, the distribution and physiological functions of these two receptors in normal tissues and tumor tissues are significantly different. DR4 is distributed and highly expressed in many immune-related tissues as well as some specific types of tumor cells, while DR5 is widely distributed in normal tissue cells at very low level but highly expressed in many different types of tumor cells (Surget et al., 2012; de Miguel et al., 2016). It was well accepted that TRAIL (Pollack et al., 2001) can transmit apoptotic signals by activating either the apoptotic receptor DR4 or DR5, however, although both the two receptors are highly expressed on the surface of a variety of tumor cells, the relative effects and mechanisms of DR4 or DR5 on apoptosis of different tumor cells, as well as the specific association between the level of receptor expression and the activation of molecular responses by DR4 or DR5 has not yet been clarified (Humphreys and Halpern, 2008), which are probably not solely determined by their surface expression but may be influenced by intracellular apoptotic regulators.

MacFarlane *et al.* had revealed that TRAIL signals to apoptosis were predominantly transmitted via DR4 in chronic lymphocytic leukemia cells as well as pancreatic carcinoma cells (Stadel et al., 2010; MacFarlane et al., 2005a; Natoni et al., 2007; MacFarlane et al., 2005b), Micheau et al. reported that DR4 as a master player of apoptosis induced by TRAIL and ER stress (Dufour et al., 2016). Meanwhile, more studies had shown that DR5 probably played a major role in the initiation of apoptosis (Ichikawa et al., 2001; Nahacka et al., 2018) and showed better potential for antitumor drug development, for the basis phenomenon that DR5 exhibits high levels of expression in plenty types of cancer cell lines while expressed very low expression in normal tissues, which indicates the potential safety advantage for tumor targeted therapy, and for that DR5 contains the highest affinity to TRAIL at the optimal human body temperature of 37°C (Truneh et al., 2000). Kelley et al. (2005) conducted apoptosis experiments comparing TRAIL variants bound solely to DR4 or DR5, and revealed that lung, colon, and breast cancer cell lines show similarity in the membrane surface expression levels of DR4 and DR5 and exhibit more significant sensitivity to specific mutants of DR5 (Kelley et al., 2005). And the preferential agonistic DR5 antibody reactivity of ovarian, colon, and renal cell carcinoma cell lines is closely related to their high surface DR5 expression levels (Zeng et al., 2006; Nawrocki et al., 2007; Saulle et al., 2007; Marini et al., 2006). These findings emphasize the need to identify TRAIL receptor subtypes that can preferentially or precisely signal apoptosis in a given type of cancer.

### 2.2 Expression of DR5

DR5 is expressed across a variety of normal human tissues such as the heart, lungs, thymus, liver, kidneys, colon, small intestine, ovaries, prostate, testes, and skeletal muscles with very low levels. In addition, it has been confirmed that (Wu, 2009) DR5 is generally expressed at extremely higher levels than in normal tissues in a variety of tumor cell types, including breast, endometrial, cervical, ovarian, pancreatic, hepatocellular, and rectal cancers. DR5 expression is most common in bone sarcomas (e.g., Ewing’s sarcoma, osteosarcoma, and chondrosarcoma) as well as hematological tumors such as myeloma (Surget et al., 2012; Picarda et al., 2010; Chen W. et al., 2021; Newton, 2023). Subbiah et al. (Newton, 2023) reported that the DR5 agonist INBRX-109 showed encouraging antitumor activity and a favorable safety profile in patients with unresectable/metastatic chondrosarcoma in a phase I study. Pishas et al. (2013) *.* Evaluated the efficacy of drozitumab, a human monoclonal agonistic antibody against DR5, as a novel therapeutic avenue for the targeted treatment of bone and soft tissue sarcomas. Because DR5 is highly expressed on the cell surface of primary osteosarcoma and soft tissue sarcoma (Gamie et al., 2024), targeting DR5 in combination with other antitumor agents has become a promising strategy for the treatment of bone tumors and soft tissue sarcomas.

Various mechanisms have been reported for DR5 upregulation, including CHOP [since CHOP acts as a dimer with other C/EBP proteins, it may form a heterodimer with C/EBPb on the DR5 promoter (Ubeda et al., 1999)]; Activation of ERK [ERK 1/2 and RSK 2 signaling leads to ATF 4 activation, which in turn promotes CHOP induction and subsequent DR 5 expression (Oh et al., 2010)]; p53 [p53 has been shown to directly transactivate the DR5 gene (Takimoto and El-Deiry, 2000)]; JNK [JNK has been shown to activate CHOP by binding to the AP-1 binding site in the CHOP promoter region, which then upregulates DR5 expression (Ubeda et al., 1999)]; Sp1 [Activated Sp1 binds the TATA-minor promoter of the DR5 gene, which contains two Sp1 binding sites spanning regions 198 to 116. Sp1 binding is important for basal transcription of DR5 (Yoshidaa et al., 2001)]; NF-κB [the p65 subunit of NF-κB was also found to be able to increase DR5 expression by binding to the first intronic region of the DR5 gene (Chen et al., 2008)]; YY1 [The transcriptional repressor YY 1 negatively regulates DR5 transcription and expression by binding to putative DNA binding sites (804–794 bp) in the DR5 promoter (Yoshidaa et al., 2001)].

Many studies had demonstrated a significantly more important role of DR5 up-expression in promoting tumor cells apoptosis than other TRAIL receptors such as DR4. Surget et al. highlighted (Surget et al., 2012) that p53 selectively enhances the sensitivity of multiple myeloma cells to apoptosis through the modulation of DR5 but not DR4. Yang et al. (2012) demonstrated that apoptosis was induced in different hepatocellular carcinoma cell lines including Hep3B, Huh-7, and HepG2, through a combination of TRAIL and 5,7-dimethoxyflavone (DMF), with a dose-responsive augmentation of DR5 protein levels, while DR4 levels were unaffected, underscoring the pivotal role of DR5 upregulation in boosting the sensitivity to TRAIL-induced apoptosis in these hepatocellular carcinoma cell lines. Horinaka et al. (2012) reported that aclarubicin (ACR), in conjunction with TRAIL, synergistically promoted apoptosis in Jurkat cells from acute lymphoblastic leukemia and A549 lung adenocarcinoma cells by significantly increasing DR5 expression. Zhou et al. (2013) investigated the impact of zingiber officinale (casticin) on H157 tumor cells apoptosis and documented a marked elevation in DR5 expression while DR4 expression remained stable. Sakai et al. (Todo et al., 2013) discovered that ibuprofen amplified TRAIL-induced apoptosis in HCT116 tumor cells by promoting DR5 expression at both the gene and protein levels while without affecting DR4 expression. Chen et al. (2012) showed that ROS-dependent and CHOP- regulated DR5 expression played a critical role in IOA synergistic enhancement of TRAIL-induced apoptosis in HepG2 cells. Kim et al. (2008) demonstrated that rosiglitazone enhanced TRAIL-induced apoptosis in a variety of cancer cells through ROS-mediated upregulation of DR5 and downregulation of c-FLIPs. Moon et al. (2013) showed that apiacein A (VA) triggered TRAIL-induced apoptosis by generating ROS in response to eIF2α/CHOP-dependent DR5 induction. Taniguchi et al. (2008) showed that in SW 480 colon cancer cells, baicalein upregulated CHOP expression, which subsequently induced DR5 expression and restored sensitivity to TRAIL-induced apoptosis while baicalein increased DR5 transcription in a reactive oxygen species (ROS)-dependent manner in T-cell leukemia Jurkat cells and prostate cancer cell lines PC 3 and DU 145.

Meanwhile, it should be specially addressed that the cellular localization regulation mechanism of DR5 is very complex and has not been fully clarified (Mert and Sanlioglu, 2017; Ren et al., 2004)^,^ the DR5 localization on the cell membrane is the prerequisite for its initiation of apoptosis, that is, only when upregulation of DR5 occurs on the cell surface is it directly associated with its pro-apoptotic effect. Haselmann et al. reported that DR5 had a dual but opposite function, that is, when bound by TRAIL on the cell surface, it can induce apoptotic cell death, but once inside the nucleus, it promotes cell survival and/or proliferation (Haselmann et al., 2014). Liu et al. showed that the isolation of esophageal cancer cells (EC9706) did not affect total DR5 protein levels in the cells, but provided a relocation of DR5 to the cell surface (Liu et al., 2009). The localization regulation of DR5 is regulated by multiple levels such as post-translational modification, vesicle transport and stress signal, and its dynamic distribution directly determine the selection and initiation of pro-apoptotic or pro-proliferative functions. In-depth understanding of the localization mechanism of DR5 can provide theoretical basis for the development of novel therapies targeting tumor apoptosis pathways, especially in the field of overcoming drug resistance and precision medicine.

### 2.3 Biological functions of DR5

**FIGURE 1:**
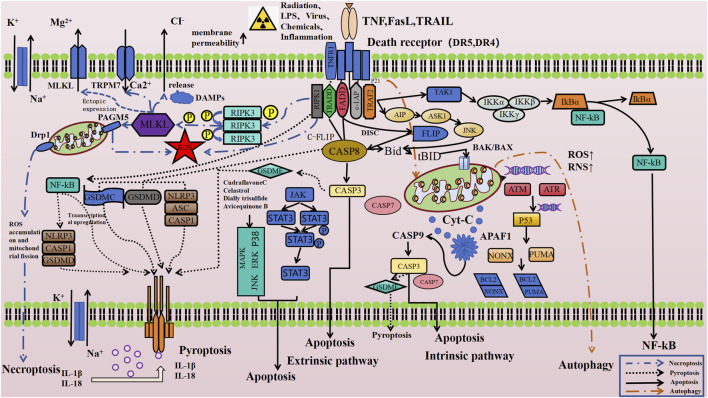
TRAIL-DR5 related cell death regulation signaling pathway (Das et al., 2017; Hu et al., 2019; Gielecińska et al., 2023; Tong et al., 2022; Peng F. et al., 2022; Sanz et al., 2023; Bertheloot et al., 2021; De Meyer et al., 2024; Hadian and Stockwell, 2023; Chen X. et al., 2021; Bedoui et al., 2020; Vandenabeele et al., 2023; Yuan and Ofengeim, 2024; Newton et al., 2021; Hou et al., 2020). Created with BioRender.com, accessed on 10 October 2024.

Mediating the classic TRAIL apoptosis signaling pathway is the core biological function of DR5 (Yang, 2012; Cotter and Al-Rubeai, 1995; Bock and Riley, 2023; Moon et al., 2023). Recent research into the complex cell death regulation mechanisms has also indicated that DR5 might also be involved in the regulation of other cell death pathways such as necroptosis (Aikawa et al., 2023; Hayashi et al., 2021; Holler et al., 2000) and autophagy (Das et al., 2017; Hu et al., 2019)-dependent cell death. Although no direct studies have yet demonstrated a link between DR5 and the regulation of pyroptosis, existing research (Fritsch et al., 2019; Orning et al., 2018; Sarhan et al., 2018) suggests that caspase-8, a key downstream molecule of the TRAIL/DR5 signaling pathway, can induce pyroptosis by cleaving GSDMD into its active form. This indirectly implies that DR5 may also play a role in the pyroptosis signaling pathway. Furthermore, our team recently found that DR5 antagonists can effectively mitigate pyroptosis-related damage in intestinal tissue cells induced by high doses of gamma radiation, both *in vitro* and *in vivo* (unpublished data). Figure 1 displayed the schematic drawing of the cell death regulatory signaling pathways that DR5 may be involved in according to the existing literature and consequent speculation.

In addition to regulating cell death, DR5 also plays key regulatory roles in a variety of physiological and pathological processes, including proliferation promotion (Belyanskaya et al., 2008; Vilimanovich and Bumbasirevic, 2008; Ishimura et al., 2006; Wang et al., 2013; Secchiero et al., 2008; Secchiero et al., 2004; Trauzold et al., 2006; Von Karstedt et al., 2017; Schneider et al., 1997; Muhlenbeck et al., 1998; Lee et al., 2002; Milani et al., 2003; Choo et al., 2006; Song and Lee, 2008; Xu et al., 2010; Liu et al., 2024), inflammation (Ehrhardt et al., 2003), tissue regeneration (Vilimanovich and Bumbasirevic, 2008)^,^ immune regulation (Ishimura et al., 2006), anti-tumor (Secchiero et al., 2008; Secchiero et al., 2004), maintenance of body development and homeostasis (Guo et al., 2005; Voigt et al., 2014; Wang, 2014), *etc.*, and intersects with a variety of other signaling pathways to form a complex regulatory network. Table 1 summarizes the main biological functions of DR5.

**TABLE 1:** Biological functions of DR5.

Functionality	Mechanism	Physiological role	Example
Apoptosis pathway	1. DR5 mediates classical apoptosis through its interaction with TRAIL, forming a trimeric complex that recruits apoptotic signaling molecules including the Fas-associated death domain protein (FADD) and Caspase-8. The activation of Caspase-8 triggers a cascade that culminates in apoptosis	1. Maintain homeostasis and normal development: as a major exogenous apoptosis regulatory pathway, DR5 has the highest affinity with TRAIL under physiological conditions and effectively promotes apoptosis of damaged cells when organism is subjected to exogenous injury	Sheridan et al. (1997), Marsters et al. (1996), Toffoli et al. (2021), Wang et al. (2021a), Wilson et al. (2012)
	2. Under some certain conditions, when DR5 is over expressed, it may directly induce apoptosis without relying on the corresponding ligand	2. Selective killing tumor cells: For that DR5 is highly expressed in a variety of tumor cells while very low expressed in normal tissues	
Non- apoptosis pathway	Participate in non-apoptotic cell death pathways	Maintain tissue homeostasis, immunomodulation and cope with environmental stress (hypoxia, oxidative stress, *etc.*)	Thon et al. (2006), Guo et al. (2005), Voigt et al. (2014)
	Under specific conditions such as caspase inhibition, oxidative stress or enhanced inflammatory signaling, DR5 may participate in the cross-regulation of other non-apoptotic cell death pathways through interaction with RIPK1, gasdermin protein or autophagy-related molecules *et al*		
	Participate in Inflammatory response: directly activate NF-κB and MAPK pro-inflammatory pathway, promote the expression of inflammatory factors and chemokines; increase the ROS level in mitochondria or promote potassium ion outflow, activate NLRP3 inflammasome and promote the secretion of IL-1β and IL-18; Promote macrophage polarization and T cell activation and indirectly regulate inflammation and immune response	1. Immune defense and host protection: When apoptosis signal is inhibited, DR5 can activate NF-κB,NLRP3 inflammasome and immune cells to enhance the clearance of pathogens	Ishimura et al. (2006), Ehrhardt et al. (2003)
		2. Participate in pathological processes of autoimmune disease and tumor progression: over-activation of DR5 leads to continuous activation of NF-κB and MAPK signals, promoting inflammatory factor storms and tissue damage; enhances immunosuppressive TAMs, MDSCs, *etc.*, Recruitment to promote tumor immune escape	
	Promote cell proliferation: Activate NF-κB pathway, promote cytokine secretion through AKT, ERK1/2, JNK, PKC pathway, induce anti- apoptotic gene (Bcl-2, c-FLIP *etc.*) expression; activate MAPK/ERK pathway, promote cyclins expression; activate PI3K/AKT pathway, inhibit proapoptotic gene (Bad, FoxO) expression	1. Tissue repair and regeneration: promote the activity of specific cells such as intestinal stem cells to maintain intestinal epitlial renewal and barrier function	Belyanskaya et al. (2008), Vilimanovich and Bumbasirevic (2008), Ishimura et al. (2006), Wang et al. (2013), Secchiero et al. (2008), Secchiero et al. (2004), Trauzold et al. (2006), Von Karstedt et al. (2017), Schneider et al. (1997), Muhlenbeck et al. (1998), Lee et al. (2002), Milani et al. (2003), Choo et al. (2006), Song and Lee (2008), Xu et al. (2010), Liu et al. (2024)
		2. Tumorigenesis and drug resistance: promotes the survival, proliferation and metastasis of tumor cells by activating the NF-κB or MAPK pathway, and leads to such as tumor immune evasion and drug resistance	

## 3 Clinical new drug development targeting DR5

### 3.1 Agonist studies related to targeting DR5

Research on agonists targeting DR5 predominantly focuses on oncology due to DR5’s overexpression in various tumor cells and rare expression in normal tissues, establishing DR5 as a significant target for tumor therapy. Additionally, DR5 agonists are investigated in autoimmune diseases, liver fibrosis, and other conditions. Current studies on DR5 agonists are summarized in Table 2.

**TABLE 2:** DR5 agonists and their applications.

Drug/candidate	Indications	Mechanism
Bioymyfi	Glioma	The pioneering small molecule agonist of DR5 uniquely activates the extrinsic apoptotic pathway, initiating tumor cell apoptosis (Wang, 2014)
INBRX-109	Unresectable/metastatic chondrosarcoma	Currently undergoing Phase I clinical trials, a third-generation recombinant humanized agonistic antibody targets DR5. This antibody achieves selective DR5 agonism, favoring the apoptosis of cancer cells while sparing normal cells (Subbiah et al., 2023; Newton, 2023; Classic, 2018a)
Dulanermin	Chondrosarcoma (medicine)	Dulanermin, the inaugural DR5 agonist utilized clinically, activates DR5-mediated apoptotic pathways (Subbiah et al., 2012; Soria et al., 2010; Soria et al., 2011; Kelley et al., 2001; Pollack et al., 2001)
IGM-8444	Solid and hematologic malignancies	A multimeric anti-DR5 IgM agonist antibody, characterized by its high-affinity binding to DR5, effectively induces apoptosis in cancer cells through the promotion of DR5 multimerization (Wang et al., 2021b; Classic, 2018b)
Surrobody	Breast cancer (BC)	Dual agonists of DR4 and DR5 have been developed that activate both receptors, initiating the apoptotic death of cancer cells, while circumventing decoy receptor involvement. These agents demonstrate notable preclinical pro-apoptotic effects (Milutinovic et al., 2016)
Recombinant human Apo2L/TRAIL (Ashkenazi et al., 2008)	Breast, colon, lung, CNS, kidney, pancreatic, and prostate cancers; leukemia, lymphoma, multiple myeloma, and non-Hodgkin’s lymphoma (NHL)	In clinical phase I/II trials, a therapeutic agent binds to both DR4 and DR5 receptors, activating the extrinsic apoptotic pathway. As this pathway functions independently of p53, the agent potentially circumvents tumor cell resistance to traditional treatments, selectively inducing apoptosis in cancer cells and sparing healthy ones (Herbst et al., 2010; Ashkenazi, 2008; Ashkenazi and Herbst, 2008; Ashkenazi et al., 2008; Ashkenazi et al., 1999; Pollack et al., 2001; Lawrence et al., 2001; Qin et al., 2001)
DR5 selective agonists	Breast and ovarian cancer (senescent cancer cells)	A DR5-selective agonist enhances exogenously induced apoptosis in senescent cancer cells (Soto-Gamez et al., 2022)
HexaBody-DR5/DR5	Multiple myeloma (medicine) (MM)	A combination of two noncompetitive DR5-specific IgG1 antibodies has shown the highest cytotoxic activity in samples from patients with relapsed/refractory multiple myeloma, particularly those previously treated with anti-MM medications, indicating a possible sensitization effect (van der Horst et al., 2021; Overdijk et al., 2019; Di Cristofano et al., 2023; Carter and Rajpal, 2022)
DS-8273a	Advanced solid tumor	A novel humanized monoclonal agonistic antibody that binds to DR5 with high affinity, is activated in tumors at the highest administered dosage (Forero et al., 2017)
TRA-8	Triple-negative breast cancer (TNBC)/rheumatoid arthritis	A DR5-specific agonistic antibody selectively induces apoptosis in malignant cells without affecting normal hepatocytes (Fancy et al., 2018) TRA-8 reduces the severity of arthritis by targeting macrophage depletion and immunomodulatory effects (Li et al., 2012)
Single-chain antibody TR2-3 combined with cisplatin	Colorectal cancer (CRC), BC	Cisplatin enhances the sensitivity of COLO205 and MDA-MB-231 cancer cells to apoptosis mediated by TR2-3, a novel agonistic single-chain antibody targeting DR5, by increasing DR5 expression (Lei et al., 2018)
Lipid carrier protein 2 (LCN2)	CRC	Also known as oncogene 24p3 or NGAL, a 25 kDa secreted glycoprotein that sensitizes TRAIL in DR-targeted CRC therapy (Kim et al., 2018)
Conatumumab (AMG 655)	CRC, non-small cell lung cancer (NSCLC)	In clinical phase I trials, a fully human monoclonal agonistic antibody (IgG1) specific to DR5, prevalent across various tumor types, swiftly triggers caspase-8 activation leading to apoptosis (Spierings et al., 2003; Maduro et al., 2009; Ganten et al., 2009; Leithner et al., 2009; McCarthy et al., 2005; Kaplan-Lefko et al., 2010)
lexatumumab (HGS-eTR2)	Advanced malignant tumor	In a similar clinical phase I study, a fully human agonist monoclonal antibody directed against TNF-associated apoptosis-inducing ligand receptor 2 efficiently activates exogenous apoptotic pathways, displaying robust antitumor activity in patients with advanced solid tumors (Plummer et al., 2007; Zhao et al., 2016)
Tigatuzumab (CS-1008)	Unresectable or metastatic pancreatic cancer	In the second phase of clinical trials, a humanized variant of the stimulating mouse monoclonal antibody TRa-8 targets DR5, initiating apoptosis across human cancer cell lines via a caspase cascade (Forero‐Torres et al., 2013; Rajeshkumar et al., 2010)
RG7386	Breast, colorectal, and lung cancer	A groundbreaking tetravalent FAP-DR5 antibody has been developed to efficiently initiate apoptosis in tumor cells in preclinical models, particularly targeting FAP-positive stromal environments (Brünker et al., 2016)
MEDI3039	lymphoma	An *ex vivo* and *in vivo* potent DR5 agonist that induces regression of *in situ* tumors and inhibits the growth of metastatic TNBCs, showing therapeutic potential in patients with BC, especially those with basal B TNBC (Greer et al., 2019)
Drozitumab	Pancreatic	An agonistic DR5 antibody selectively targets CSCs, inhibiting tumor growth and even causing regression in pancreatic tumors (Eng et al., 2016)
rhTRAIL or Drozitumab (both clinically evaluated) in combination with a histone deacetylase inhibitor (HDACi)	Melanoma (type of skin cancer)	Both rhTRAIL (Dulanermin) and Drozitumab were clinically evaluated in combination with the histone deacetylase inhibitor (HDACi) SAHA to lower the apoptotic threshold and achieve better clinical outcomes in melanoma (Pollack et al., 2001; Yang et al., 2010; Lim et al., 2013; Kang et al., 2011; Adams et al., 2008)
Recombinant human TRAIL and drozitumab	Melanoma (type of skin cancer)	Recombinant human TRAIL combined with drozitumab confirms the efficacy of the DR5/TRAIL pathway in eliminating melanoma cells (Jazirehi et al., 2014)
TAS266	Advanced solid tumor	In clinical phase I, TAS266 potentially raises DR5 expression on hepatocytes, augmenting the effectiveness of Nanobody by promoting enhanced DR5 clustering and hepatocyte apoptosis activation (Papadopoulos et al., 2015)
Agonistic DR5 antibody D-6 combined with cisplatin	Ovaries	The agonistic DR5 antibody D-6, a novel candidate for combating C30 cisplatin-resistant ovarian cancer, initiates apoptosis through both cysteine-dependent and independent pathways and may help reduce cisplatin resistance in the C30 cell line (Huang et al., 2014)
DS-8273a	Leukemia, MM, melanoma, breast, bladder, prostate, kidney and colon cancers	Currently in clinical phase I, it is an anti-human DR5 agonistic antibody that induces apoptosis upon specific binding to DR5 (Voigt et al., 2014; Herbst et al., 2010; Burvenich et al., 2016; Dominguez et al., 2017)
Drozitumab	Lymphoma	A fully human monoclonal antibody targets DR5 and effectively induces apoptosis (Zinonos et al., 2014)
Novel TRPV1 antagonist DWP05195	Human ovarian cancer	TRPV1, a non-selective ligand-gated cation channel with high calcium permeability, is activated by various stimuli, including low pH, extreme heat, and vanilloids (Szallasi et al., 2007). The agent DWP05195 induces apoptosis in human ovarian cancer cells by activating endoplasmic reticulum stress via the ROS-p38-CHOP pathway (Wang et al., 2020a)
Parthenolide (PTL)	Human oral cancer	Strongly induces apoptosis and enhances DR5 protein expression, associated with increased cysteinyl asparagine-8 cleavage and Bid (t-Bid) truncation (Yu et al., 2015)
Mapatumumab	Advanced solid malignant tumor	A humanized IgG2 monoclonal antibody targeting TRAIL-R1 that binds DR5 and activates the apoptotic pathway, currently in clinical trials including combination therapies (Maddipatla et al., 2007)
Epinephrine (Shaite)	Relapsed Refractory MM (RRMM)	Eponemin, a self-developed Class 1 innovative drug, acts as an agonist for DR4/DR5. It binds and activates these receptors on the surface of tumor cells, initiating the intracellular caspase cascade via the extrinsic apoptosis pathway, thereby exerting antitumor effects (Author Anonymous, 2025a; Author Anonymous, 2025b)
Zaptuzumab	Bone tumors and soft tissue sarcomas	Zaptuzumab is a fully humanized agonist of DR5 (zaptuzumab) linked to a toxic inhibitor of tubulin, monomethyl auristatin D (MMAD) (Zhang et al., 2019; Zheng et al., 2023)
ABBV-621	Patients with advanced solid tumors, hematologic malignancies	ABBV-621 is a second-generation TRAIL-R agonist that selectively binds to DR5 to drive targeted biological activity with potent caspase-dependent antitumor activity that enhances caspase-8 aggregation, death-induced signaling complex formation, and is independent of Fc-FcγR interactions (LoRusso et al., 2022; Phillips et al., 2021)
BI 905711	Colon cancer, gastrointestinal tract cancer, pancreatic cancer	BI 905711 is a tetravalent bispecific antibody targeting TRAILR2 and CDH17, which effectively initiates apoptosis cascade upon binding to DR5, strongly increases caspase-8 and caspase-3/7 aggregation, and death-induces signaling complex formation (Garcia-Martinez et al., 2019; Harding et al., 2023; Han et al., 2021)
Troglitazone	Malignant tumor	TGZ enhances DR5 expression at the promoter level through the CCAAT/enhancer binding protein homologous protein (CHOP) binding site (Koyama et al., 2014)
Apomab	Heterogeneous colon or pancreatic tumors	Apomab is a DR5 agonist at one of the TRAIL receptors (Huang et al., 2023)

### 3.2 Antagonist studies targeting DR5

Compared to DR5 agonists, research into DR5 antagonists commenced later. However, as studies on DR5-mediated cell signaling pathways deepened. The researchers (Leng et al., 2014) found that DR5 is over-activated in response to external stimuli leading to excessive cell death and impaired function in target organs and tissues, which points to a new direction in exploring the development and pathological mechanisms of various clinical diseases. Currently, the focus on DR5 antagonists has intensified, showing significant promise for treating conditions associated with DR5 hyperactivation, such as severe viral infections (Peng H. et al., 2022), inflammation, ischemia-reperfusion injury (Xiaochun et al., 2022; Zhang, 2018; Liu, 2018), and autoimmune diseases. Our team’s studies have also shown that high-dose γ-rays (Zhao et al., 2023) significantly increase DR5 expression in vital organs and tissues, and administering DR5 antagonist interventions notably improved survival rates and organ function recovery in animals with acute radiation sickness. Research and development efforts for DR5 antagonists currently encompass the screening of small molecule compounds, antibody structures, and peptide sequence optimization. Current studies targeting DR5 antagonists are outlined in Table 3.

**TABLE 3:** DR5 antagonists and their applications.

Drug/candidate	Indications	Mechanism
Soluble sDR5	Acute Kidney Injury (AKI)	Soluble DR5 therapy reduces apoptosis and attenuates burn-induced kidney injury by inhibiting endogenous TRAIL-DR5 interaction (Leng et al., 2014)
	Acute hepatitis	sDR5 reduces liver injury by mitigating TRAIL-induced apoptosis in HBV-infected hepatocytes (Liu et al., 2007)
Soluble DR5 Fusion Protein (sDR5-Fc)	Acute radiation syndrome (ARS)	A competitive antagonist of DR5 effectively reduces excessive apoptosis in radiation-sensitive tissues like the spleen and thymus, diminishes radiation-induced damage to these organs, and significantly enhances the expression of apoptosis-inhibitory proteins such as Bcl-2 (Zhao et al., 2023)
	Coronavirus or chronic metabolic diseases	Soluble DR5-Fc fusion proteins significantly reduced SARS-CoV-2-induced inflammatory responses by blocking TRAIL-DR5 interactions (Peng et al., 2022b)
	Acetaminophen (APAP)-induced liver injury	The use of sDR5-Fc significantly curtails APAP-induced liver injury, hepatic leukocyte infiltration, inflammatory cytokine production, and mortality in mice. This effect is amplified when sDR5-Fc is used alongside N-acetylcysteine, offering heightened protection against APAP-induced acute liver injury (Chen et al., 2020)
	Cardiac ischemia-reperfusion (I/R) injury (Xiaochun et al., 2022)	sDR5-Fc mitigates myocardial I/R injury by inhibiting peripheral blood neutrophil infiltration. And reducing macrophage-mediated inflammatory responses (Zhang, 2018; Liu, 2018)

## 4 Current status, problems, and trends of clinical drug research and development targeting DR5

**FIGURE 2:**
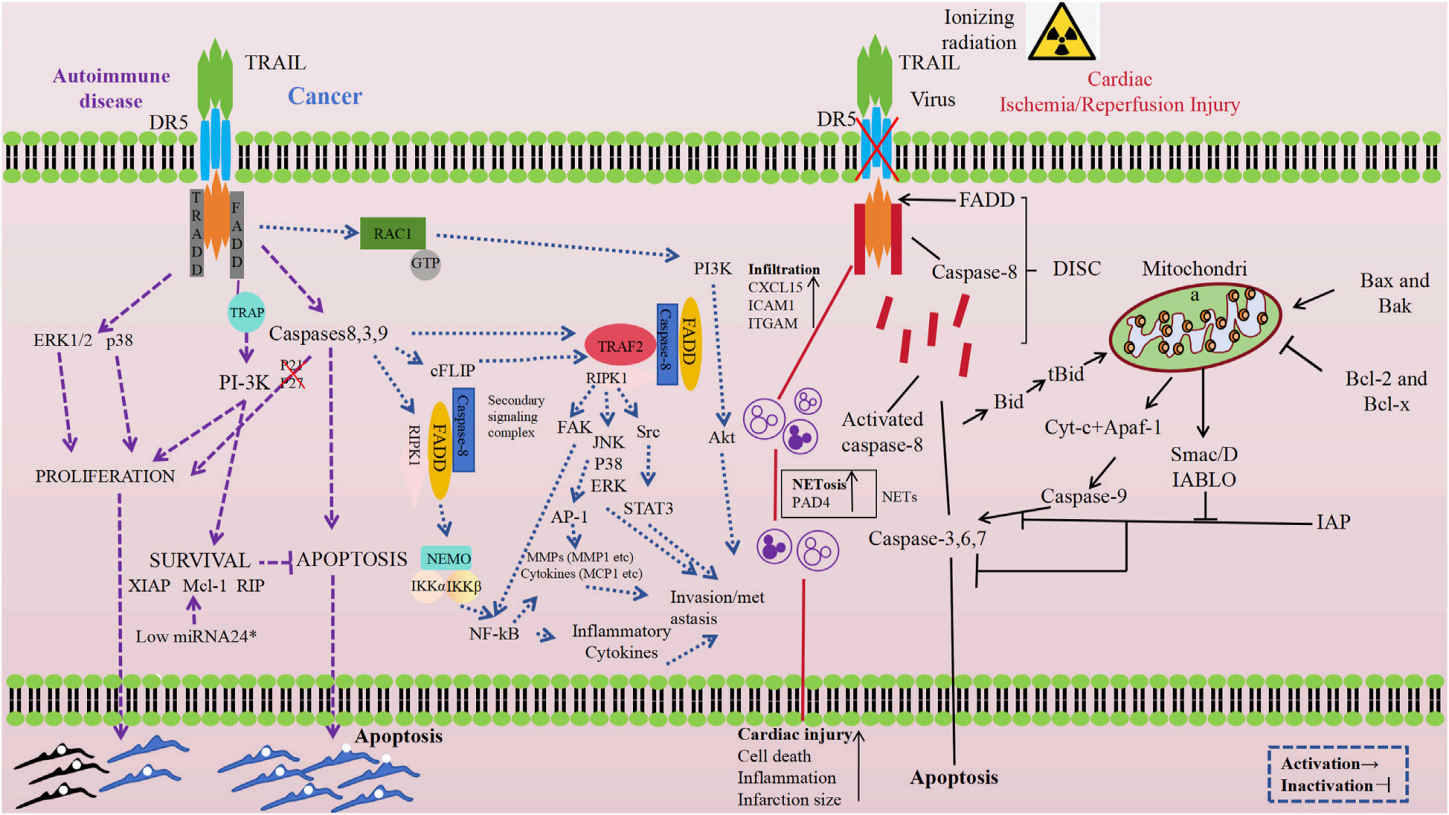
Schematic representation of DR5 antagonists or agonists effects in different diseases (Peng H. et al., 2022; Zhang, 2018; Collison et al., 2009; Oh and Sun, 2021; Audo et al., 2013; Wang et al., 2024). Created with BioRender.com, accessed on 10 October 2024.

With advancing research, the TRAIL-DR5 pathway has been recognized for its crucial physiological functions in maintaining normal physiological homeostasis and growth. A growing body of evidence suggests that it plays an important role in the onset and progression of a variety of diseases, with the specific mechanisms described in Figure 2. External stimuli can significantly upregulate DR5 protein expression, activating the TRAIL-DR5 signaling pathway and triggering apoptosis. While this activation is a key regulatory mechanism for homeostasis, it may also cause excessive death of functional cells in target organs in some instances. Numerous studies have explored the efficacy and safety of targeting DR5 in various diseases using activation or antagonism strategies, analyzing potential challenges and future research directions.

### 4.1 Tumor targeting therapy

DR5 has been extensively explored as a novel drug target in oncology, primarily inducing apoptosis in tumor cells, and several DR5 agonists have reached clinical trials (Voigt et al., 2014; Classic, 2018a; Herbst et al., 2010; Ashkenazi, 2008; Ashkenazi and Herbst, 2008; Ashkenazi et al., 2008; Ashkenazi et al., 1999; Pollack et al., 2001; Lawrence et al., 2001; Qin et al., 2001; Burvenich et al., 2016; Dominguez et al., 2017), however, due to the phenomenon of drug resistance caused by tumor immune escape, insufficient drug delivery efficiency, and low targeting efficiency or low receptor cross-linking efficiency, the tumor targeting therapy of DR5 agonists has encountered many difficulties. At present, the research on the mechanism of drug resistance (Kim et al., 2018; Gupta et al., 2013) of DR5 agonists has attracted much attention, and the future research on DR5 agonists as tumor targeted therapy will focus on the novel DR5 agonists design (Schneider et al., 2010; Li et al., 2024), combination therapy (Zheng et al., 2023; Casagrande Raffi et al., 2024) and new delivery system development, etc., aiming to improve targeted efficacy and safety.

For example, Casagrande Raffi et al. (2024) suggested that salinomycin may be effective when used in combination with age-delaying cancer therapies. The combination of a death receptor 5 agonist antibody and Salinomycin is a potent anti-aging drug cocktail and the combination triggers immune destruction of senescent cancer cells mediated by natural killer cells and CD 8 + T cells with the involvement of interleukin-18. Li et al. (2024) prepared a coupling agent containing multiple copies of DR5-targeting peptide (P-cDR5), which significantly improved DR5 aggregation and effectively induced apoptosis. Combining P-cDR5 with the histone deacetylase inhibitor valproic acid further enhances apoptosis-inducing efficacy by increasing Caspase-8 and activating the exogenous apoptotic pathway, while destabilizing mitochondrial membranes and increasing the sensitivity of TRAIL-resistant cells. These findings suggest that ligating multiple cDR5 peptides onto flexible water-soluble polymer carriers not only overcomes the limitations of previous designs, but also provides new ideas for the treatment of drug-resistant cancers.

### 4.2 Autoimmune diseases

#### 4.2.1 Rheumatoid arthritis

DR5 agonists play a role in rheumatoid arthritis primarily through their regulatory effects on apoptosis, immunomodulation, and inflammatory responses. I-Tsu et al. (Chyuan et al., 2018) observed that DR5 activation reduced joint inflammation and destruction in a mouse model of rheumatoid arthritis. Jin et al. (2010) demonstrated that activating DR5 could induce apoptosis in synoviocytes and inflammatory cells, reducing the production of inflammatory mediators. Although DR5 agonists show potential in RA, their specific mechanisms require further investigation. Additionally, genotypic and phenotypic variations among patients may affect responses to DR5 agonists. Future research will focus on developing DR5 agonists to promote apoptosis in inflammatory cells, thereby diminishing inflammation. Furthermore, combining DR5 agonists with other anti-inflammatory or immunomodulatory drugs, such as DMARDs (Singh, 2022) or biologics, could enhance therapeutic outcomes.

#### 4.2.2 Inflammatory bowel disease

DR5 agonists regulate apoptosis and immunomodulation in inflammatory bowel disease. Zhu et al. (2014) found that DR5 activation mitigated intestinal inflammation and damage in a mouse model of IBD. *In vitro*, DR5 agonists reduced the release of inflammatory mediators by inducing apoptosis in inflammatory and intestinal epithelial cells (Kuo et al., 2019). However, the ability of DR5 to induce apoptosis in inflammatory cells does not preclude these cells from evading immune surveillance due to complex immune escape and response mechanisms. Future research is directed toward developing novel DR5 agonists that promote apoptosis in inflammatory cells to reduce inflammation. Therapeutic strategies combining DR5 agonists with other anti-inflammatory agents, such as glucocorticoids, immunosuppressants, or biologics, are also being explored to enhance therapeutic effects.

#### 4.2.3 Systemic lupus erythematosus

DR5 agonists regulate apoptosis and immunomodulation in SLE, (Hilliard et al., 2001; Song et al., 2000; Ichikawa et al., 2003), with Lamhamedi-Cherradi SE et al. (Lamhamedi-Cherradi et al., 2003; Liu et al., 2003) finding that DR5 activation reduced inflammation and organ damage in a mouse model of SLE. *In vitro* studies show that activation of DR5 can induce apoptosis in immune cells, thereby reducing inflammatory mediator production (Crowder et al., 2011). Although DR5 agonists show promise for treating SLE, no large-scale clinical trials have yet been conducted, and issues such as adverse effects and immune escape persist. Further studies is necessary to uncover the roles and influencing factors of DR5 in SLE and to examine the potential benefits of combining it with other anti-inflammatory agents to boost therapeutic effectiveness.

### 4.3 Cardiovascular diseases

Excessive cardiac cell death, a primary pathological feature of myocardial infarction (MI), can be substantially alleviated by inhibiting TRAIL with DR5 antagonists, such as sDR5-Fc fusion proteins, which have been shown to improve outcomes following myocardial infarction (MI). Wang Y. et al. (2020) noted increased levels of both DR5 and TRAIL in MI contexts, with a corresponding reduction in cardiomyocyte death and inflammation upon blocking TRAIL. Similarly, Cui et al. (2010) observed that blocking the TRAIL-DR5 interaction with a soluble DR5 antagonist decreased ischemic cell death following global cerebral ischemia, suggesting a potential neuroprotective strategy for ischemic stroke through inhibition of the TRAIL-DR5 system. Future research will focus on developing novel DR5 antagonists with improved drug properties and considering co-administration with other cardiovascular therapeutics to enhance efficacy.

### 4.4 Viral hepatitis

Research into the use of DR5 as a therapeutic target for viral hepatitis presents mixed outcomes. Mundt et al. (2003) demonstrated that apoptosis in virally infected hepatocytes is facilitated by the TRAIL-DR5 pathway, enabling the selective elimination of virus-infected hepatocytes while sparing normal cells, proposing DR5 agonists as a viable treatment for viral hepatitis. In contrast, Liu et al. (2007) reported that an sDR5 antagonist could reduce liver damage by blocking TRAIL-induced apoptosis in HBV-infected hepatocytes, underscoring the complexity and crucial role of the TRAIL-DR5 system in the pathogenesis of viral hepatitis. These findings necessitate further research to elucidate TRAIL-DR5 regulatory mechanisms in viral hepatitis, distinguish between the expression levels in virus-infected versus normal cells, and ensure the safety and efficacy of both DR5 antagonists and agonists.

### 4.5 Liver fibrosis

Liver fibrosis is closely related to DR5, an apoptosis factor receptor predominantly expressed on the surface of activated hepatic stellate cells (HSCs), which are central to the development of liver fibrosis. Studies have shown that anti-DR5 antibodies induce apoptosis in activated HSCs, exhibiting anti-fibrotic effects and presenting a potential therapeutic approach for liver fibrosis (You et al., 2021). Furthermore, TRAIL, a member of the TNF family, interacts with HSCs during both progression and reversal stages of liver fibrosis. This interaction, coupled with increased DR5 expression on HSCs, inhibits collagen formation and extracellular matrix (ECM) deposition, mitigating liver fibrosis (Tao et al., 2021). Additional research indicates that exogenous TRAIL induces apoptosis in activated HSC-T6 cells, potentially through upregulated DR5 and mitochondrial Bax expression (Gao et al., 2010). These insights highlight the pivotal role of DR5 in liver fibrosis, suggesting that modulation of DR5 expression and function could be a strategy to control or reverse hepatic fibrosis. However, the mechanisms by which DR5 operates and its potential clinical applications in liver fibrosis are still largely unexplored; future studies should aim to detail the specific regulatory mechanisms of TRAIL-DR5 in liver fibrosis and the differential expression between fibrotic and healthy liver cells to improve the safety and effectiveness of DR5-targeted therapies.

### 4.6 Radiation damage

The potential of DR5 antagonists in treating acute radiation damage has recently begun to be investigated. Our team’s previous research (Zhao et al., 2023) had shown that antagonizing DR5 significantly enhanced survival rates in an acute radiation sickness mouse model, reduced tissue damage as well as inflammatory responses, and inhibited the excessive apoptosis of functional cells. And our further study indicated that DR5 antagonist efficiently inhibited the enterocytes excessive pyroptosis caused by large dose of γ-radiation (unpublished data), which indicated the great potential of DR5 antagonist as radiation damage protection candidate. However, the comprehensive cellular regulatory mechanisms of DR5 under biological damage effects caused by radiation require further investigation. Future studies should focus on the regulatory mechanism of cell death, designing novel DR5 antagonists, optimizing their metabolic properties *in vivo* and addressing the key safety concerns, etc.

## 5 Summary

In conclusion, although the exploration of DR5 in disease treatment is nascent, its potential therapeutic benefits and broad applicability are promising. Future research should develop diverse, precise, and combinatorial therapeutic strategies based on an in-depth understanding of DR5’s biological activity and regulatory mechanisms, to extend its application across various disease treatments.
